# When the Position of Pendant Groups Makes the Difference in G‑Quadruplex Behavior: The Case of Bis-Conjugated Thrombin-Binding Aptamers

**DOI:** 10.1021/acs.jcim.5c01598

**Published:** 2025-12-24

**Authors:** Chiara Platella, Federica Battistini, Claudia Riccardi, Michael Smietana, François Morvan, Modesto Orozco, Daniela Montesarchio

**Affiliations:** † Department of Chemical Sciences, 9307 University of Naples Federico II, 80126 Naples, Italy; ‡ Institute for Research in Biomedicine (IRB Barcelona), The Barcelona Institute of Science and Technology, 08028 Barcelona, Spain; § Department of Biochemistry and Biomedicine, University of Barcelona, 08028 Barcelona, Spain; ∥ Institut des Biomolécules Max Mousseron, Université de Montpellier, CNRS, ENSCM, 34293 Montpellier, France

## Abstract

In the search for effective and low-toxicity anticoagulant agents, the G-quadruplex-forming thrombin-binding aptamer (TBA) with sequence 5′-GGTTGGTGTGGTTGG-3′, able to selectively recognize the fibrinogen-binding exosite I of the thrombin enzyme, emerged as a promising therapeutic and surgical tool. In this frame, we recently synthesized and evaluated a library of TBA analogues carrying a naphthalene diimide (**N**) moiety and a 3-hydroxypropylphosphate (**p**) either at the 5′- or 3′-end of the TBA sequence. Interestingly, **N-TBA-p** and **p-TBA-N** analogues, having the same pendant groups at 5′- or 3′-end but in reversed position, showed very different behavior in terms of thermal stability, nuclease resistance in serum, and anticoagulant activity. **N-TBA-p** showed enhanced properties compared to both **p-TBA-N** and the parent TBA and thus emerged as a very promising candidate for future in vivo studies. Here, by in-depth molecular dynamics-based analyses, we disclosed the structural features determining the higher thermal stability and nuclease resistance as well as the higher anticoagulant activity due to thrombin recognition, experimentally observed for **N-TBA-p** than **p-TBA-N** and TBA.

## Graphical abstract

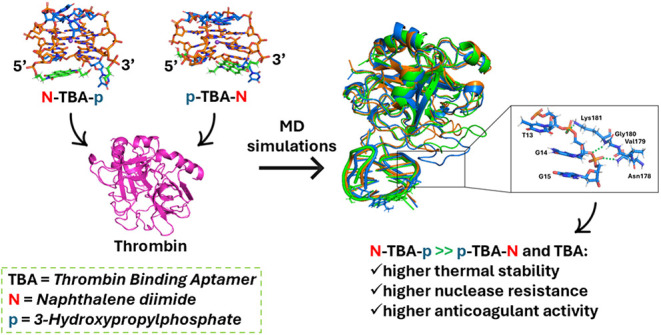

## 1 Introduction

The thrombin-binding aptamer TBA, of sequence 5′-GGTTGGTGTGGTTGG-3′, is one of the most studied aptamers due to its high application potential in both therapy and diagnostics of coagulation diseases as well as in surgery.^1–5^ In detail, TBA, being a G-rich oligonucleotide, folds into a G-quadruplex structure,^6–8^ able to adopt a chair-like antiparallel conformation, which can recognize the fibrinogen-binding exosite I of thrombin thus interfering with the last step of the coagulation cascade.^9,10^

Although TBA evidenced a promising pharmacokinetic profile in humans, its preclinical and clinical evaluations were halted after phase I studies due to suboptimal dosing profiles,^11–13^ which encourage the development of improved derivatives.^14^ In this context, some of us recently synthesized a library of TBA analogues (Figure S1) carrying a naphthalene diimide (**N** or **NDI**)^15–17^ moiety either at the 5′- or 3′-end (**N-TBA** and **TBA-N**) and a 3-hydroxypropylphosphate (**p** or **HPP**) group either at the other end (**N-TBA-p** and **p-TBA-N**, Figure 1) or directly attached to the naphthalene diimide (**pN-TBA** and **TBA-Np**).^18^ To further investigate the effect of the 3-hydroxypropylphosphate, the analogue of **TBA-N** with this pendant group at each end (**p-TBA-Np**), and an analogue in which the terminal hydroxyl group was removed (**TBA-NC3**) were also prepared. As a control, the TBA analogue carrying the 3-hydroxypropylphosphate group at both ends (**p-TBA-p**) was also synthesized.^18^

**Figure 1:**
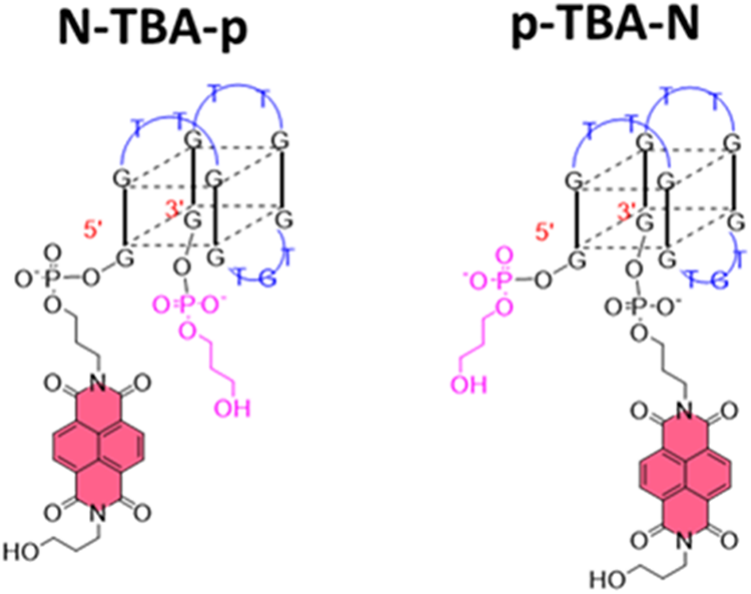
Representations of the structures of the TBA analogues here investigated: **N-TBA-p** (left) and **p-TBA-N** (right). **N** = naphthalene diimide; **p** = 3-hydroxypropylphosphate. Adapted with permission from ref 18 Copyright 2023 Elsevier.

The behavior of these TBA analogues was evaluated in a buffer mimicking the extracellular environment (Na^+^-rich buffer with approximately 150 mM sodium concentration), i.e., where the TBA and its analogues exert their anticoagulant activity, inhibiting the thrombin-catalyzed fibrinogen-fibrin conversion. The majority of the analogues have proven to be more potent thrombin inhibitors than the parent TBA. Particularly, **N-TBA-p** exhibited enhanced G-quadruplex thermal stability, improved nuclease resistance in serum, as well as higher anticoagulant activity than TBA and the highest clotting times compared to the best TBA analogues previously investigated,^19–21^ thus proving to be a very promising candidate for future in vivo studies.^18^

Interestingly, despite their similarities, **N-TBA-p** and **p-TBA-N** (Figure 1) were featured by very different properties, with **N-TBA-p** exhibiting excellent pharmacological properties while **p-TBA-N** showing one of the poorest profiles in the series.^18^ Indeed, UV- and circular dichroism-melting experiments showed that **N-TBA-p** and **p-TBA-N** featured a totally different thermal stability in Na^+^ buffer.^18^ While **p-TBA-N** showed a similar thermal stability to the parent TBA, **N-TBA-p** was more stable than **p-TBA-N** and TBA by more than 10 °C. Tested for resistance to nuclease digestion in serum, **N-TBA-p** was found to be 3- and 10-fold more resistant than **p-TBA-N** and TBA, respectively. Moreover, when analyzed by native gel electrophoresis in Na^+^ buffer, **N-TBA-p** showed a higher electrophoretic mobility than **p-TBA-N**.^18^ Additionally, **N-TBA-p** and **p-TBA-N** gave very different properties also concerning their ability to inhibit the thrombin activity, with **p-TBA-N** having a very similar anticoagulant activity as the parent TBA, while **N-TBA-p** showing more than 7 times higher anticoagulant activity.^18^

Intrigued by these results, we here used molecular dynamics (MD) simulations to explore at the atomic level the origin of the differential stabilizing effects of naphthalene diimide and 3-hydroxypropylphosphate substituents on the TBA G-quadruplex motif and the associated improvement in pharmacological properties. Moreover, MD simulations were exploited to study **N-TBA-p** and **p-TBA-N** in their interaction with thrombin and unveil the structural features that could explain why the two aptamers are so different in terms of their inhibitory activity toward the protein.

## 2 Methods

### 2.1 Molecular Dynamics Simulations

The structures of **N-TBA-p** and **p-TBA-N** were prepared using as a starting point the crystal structure of TBA bound to thrombin in the presence of sodium ions (PDB ID 4DIH) and then covalently linking to it the naphthalene diimide and 3-hydroxypropylphosphate pendant groups.

The thrombin structure was prepared starting from the protein in PDB 4DIH and adding the missing amino acids (148–155 and 258) using the protein PDB 1MUE as template by exploiting SWISS-MODEL, a protein structure homology-modeling server.^22^

The numbering of the thrombin amino acids throughout the text is based on the following full protein sequence: ADCGLRPLFEKKSLEDKTERELLESYIIVEGSDAEIGMSPWQVMLFRKSPQELLCGASLISDRWVLTAAHCLLYPPWDKNFTENDLLVRIGKHSRTRYERNIEKISMLEKIYIHPRYNWRENLDRDIALMKLKKPVAFSDYIHPVCLPDRETAASLLQAGYKGRVTGWGNLKETWTANVGKGQPSVLQVVNLPIVERPVCKDSTRIRITDNMFCAGYKPDEGKRGDACEGDSGGPFVMKSPFNNRWYQMGIVSWGEGCDRDGKYGFYTHVFRLKKWIQKVIDQFG.

All analyzed systems were solvated in a truncated octahedral box using the TIP3P water model,^23^ with water molecules extending 10 Å from **N-TBA-p** and **p-TBA-N** or 15 Å from the aptamer/thrombin complexes. Na^+^ ions were added to neutralize the net charge and extra Na^+^ and Cl^–^ ions were added to mimic the physiological concentration of 150 mM. Parmbsc1 force field was used for the aptamers,^24^ GAFF parameters^25^ for the naphthalene diimide and 3-hydroxypropylphosphate pendant groups, ff14SB force field^26^ for thrombin, and Joung and Cheatham parameters for Na^+^ and Cl^–^ ions.^27^ Parameters and topology files for the pendant groups were prepared using Acpype.^28,29^ The systems were optimized using standard procedures involving energy minimizations, thermalization, and a final reequilibration for 10 ns,^30–32^ before performing the 1 μs unrestrained MD simulations.

For the aptamer/thrombin systems, considering the highly conservative binding mode of TBA and its analogues to thrombin,^33^ each complex was built by placing **N-TBA-p** and **p-TBA-N** at the same distance from thrombin. Then, 100 ns of restrained equilibration were run using as restraints the distances between the following atom couples as found in the case of the TBA/thrombin system (PDB entry 4DIH): O2 of T3/OE1 of Glu99, O2 of T4/ND2 of Asn101, O2 of T12/OH of Tyr98, and O4̀' of G14/NE of Arg97. After restrained equilibration, 1 μs of unrestrained MD simulation was performed for each system. In parallel, after an unrestrained equilibration step, the TBA/thrombin system was subjected to 1 μs of unrestrained MD simulation as well.

Simulations were performed using Amber18 ^34^ under isothermal–isobaric conditions (T = 298 K, P = 1 atm). For each investigated system, three MD replica copies were run. All trajectories were processed by using the CPPTRAJ module of the AmberTools18 package. Interactions were evaluated by LigPlot+.^35^ Interaction energies were estimated using classical molecular interaction potentials based on the Poisson–Boltzmann approach, accounting for both electrostatic and van der Waals contributions,^36^ and reported as average on the three MD replica copies. Figures were drawn with the aid of VMD,^37^ UCSF Chimera,^38^ PyMOL,^39^ and LigPlot+.^35^

## 3 Results and Discussion

### 3.1 MD Simulations on N-TBA-p and p-TBA-N

As described above, a bulk of experimental evidence suggests that **N-TBA-p** is more stable to thermal unfolding and much more resistant to nuclease degradation than **p-TBA-N** in physiological conditions. To gain insight into the structural features determining these high differences between the two aptamers, **N-TBA-p** and **p-TBA-N** were subjected to 1 μs of unrestrained MD simulations in the presence of sodium ions (Figures 2 and 3). Along each simulation, both aptamers showed structural stability with no major conformational changes, as inferred from the average RMSD compared to the starting structure of about 2–3 Å (Figure 4 and Table S1).

**Figure 2:**
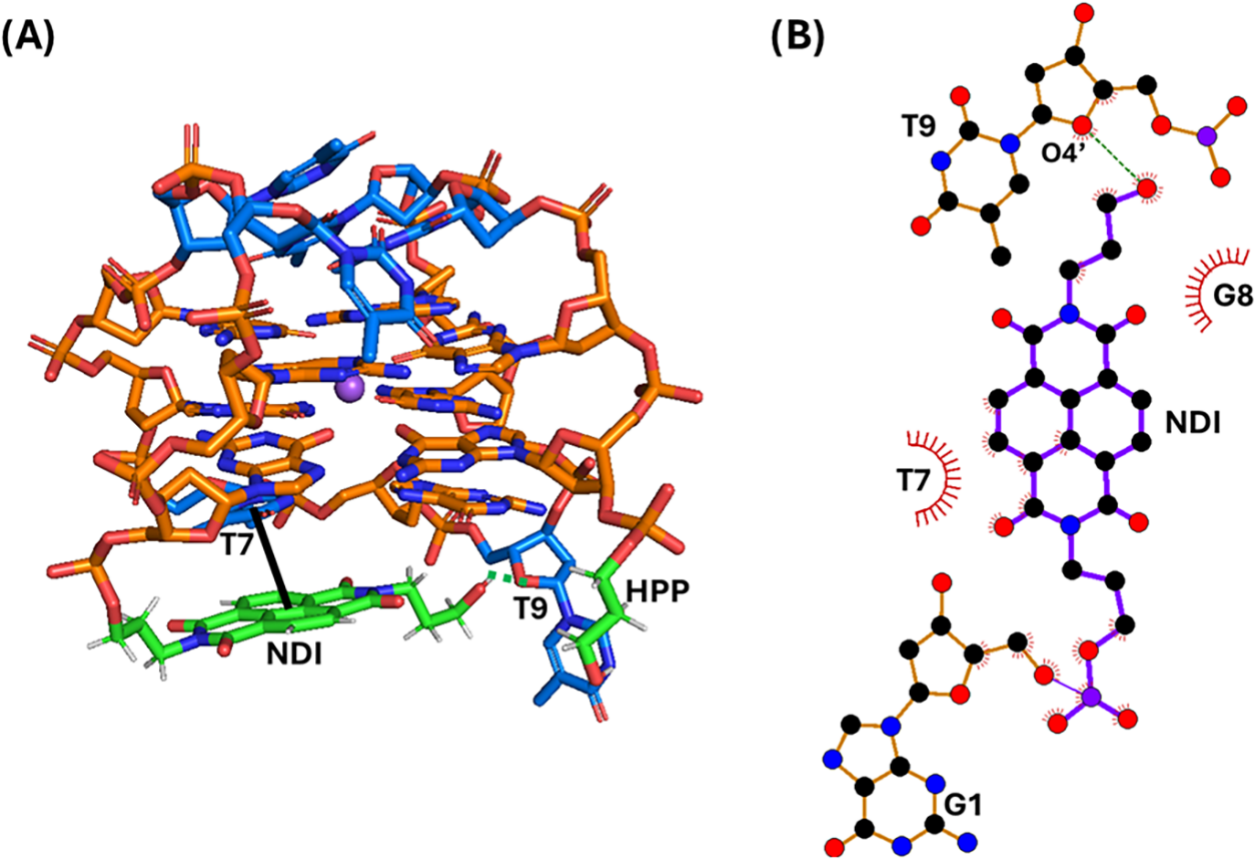
(A) Snapshot from the last frame of the 1 μs MD simulation performed for **N-TBA-p** (replica 1; for the overlapping of the three replicas, see Figure S2). The G-quadruplex aptamer is shown as a stick, while Na^+^ ion is represented as a purple sphere. Thymidines, guanosines, and the naphthalene diimide (NDI) and 3-hydroxypropylphosphate (HPP) pendant groups are colored in blue, orange, and green, respectively. Stacking interactions and hydrogen bonds are shown as black bold and green dashed lines, respectively. Nucleotides involved in the interactions with the NDI are labeled. (B) 2D interaction map. C, N, and O atoms are reported in black, blue, and red, respectively. Hydrogen atoms are not depicted for ease of illustration. Hydrogen bonds and hydrophobic contacts are depicted as green dashed lines and red arcs with radiating lines, respectively. Nucleotides and atoms involved in the interactions with the NDI are labeled.

**Figure 3:**
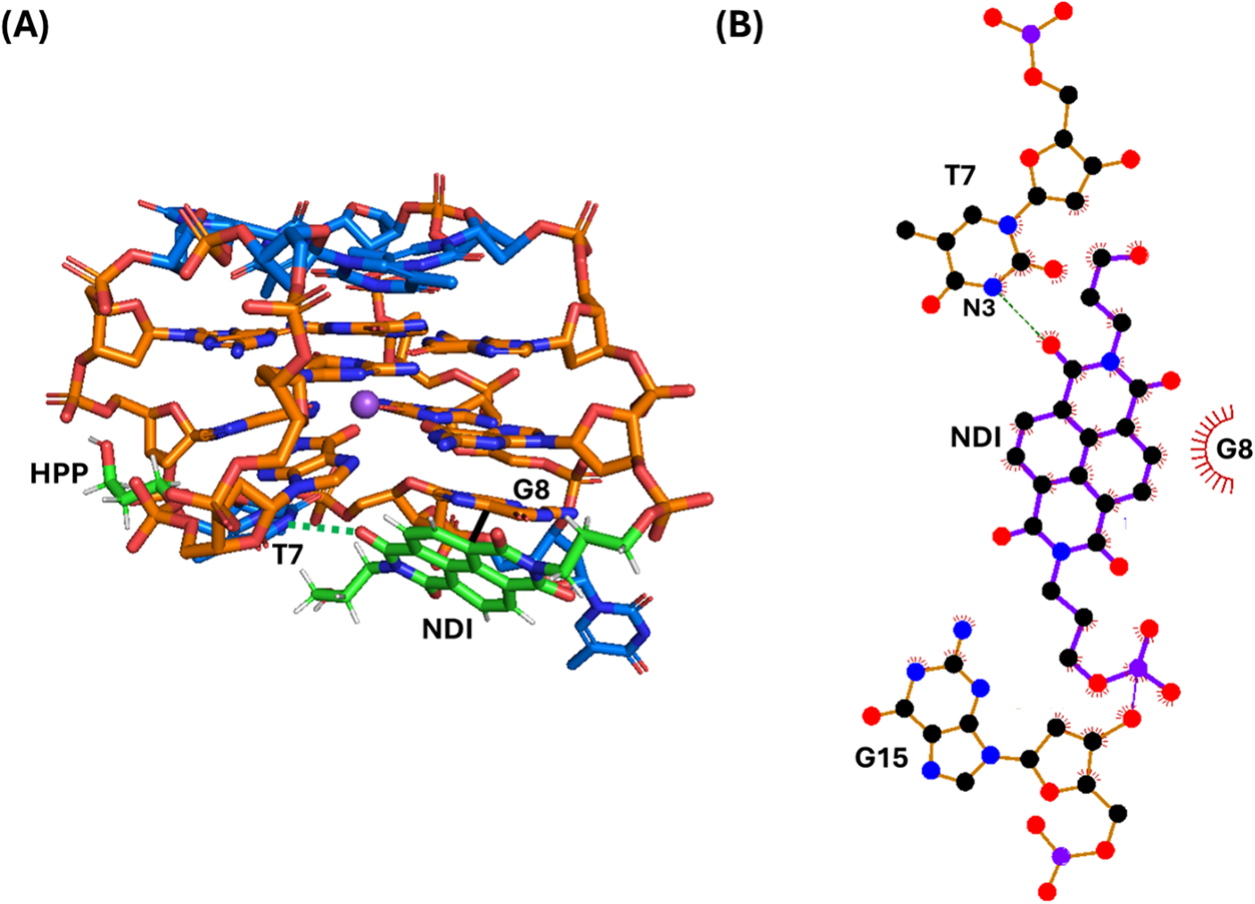
(A) Snapshot from the last frame of the 1 μs MD simulation performed for **p-TBA-N** (replica 1; for the overlapping of the three replicas, see Figure S3). The G-quadruplex aptamer is shown as stick, while Na^+^ ion is represented as a purple sphere. Thymidines, guanosines, and the naphthalene diimide (NDI) and 3-hydroxypropylphosphate (HPP) pendant groups are colored in blue, orange, and green, respectively. Stacking interactions and hydrogen bonds are shown as black bold and green dashed lines, respectively. Nucleotides involved in the interactions with the NDI are labeled. (B) 2D interaction map. C, N, and O atoms are reported in black, blue, and red, respectively. Hydrogen atoms are not depicted for ease of illustration. Hydrogen bonds and hydrophobic contacts are depicted as green dashed lines and red arcs with radiating lines, respectively. Nucleotides and atoms involved in the interactions with the NDI are labeled.

**Figure 4:**
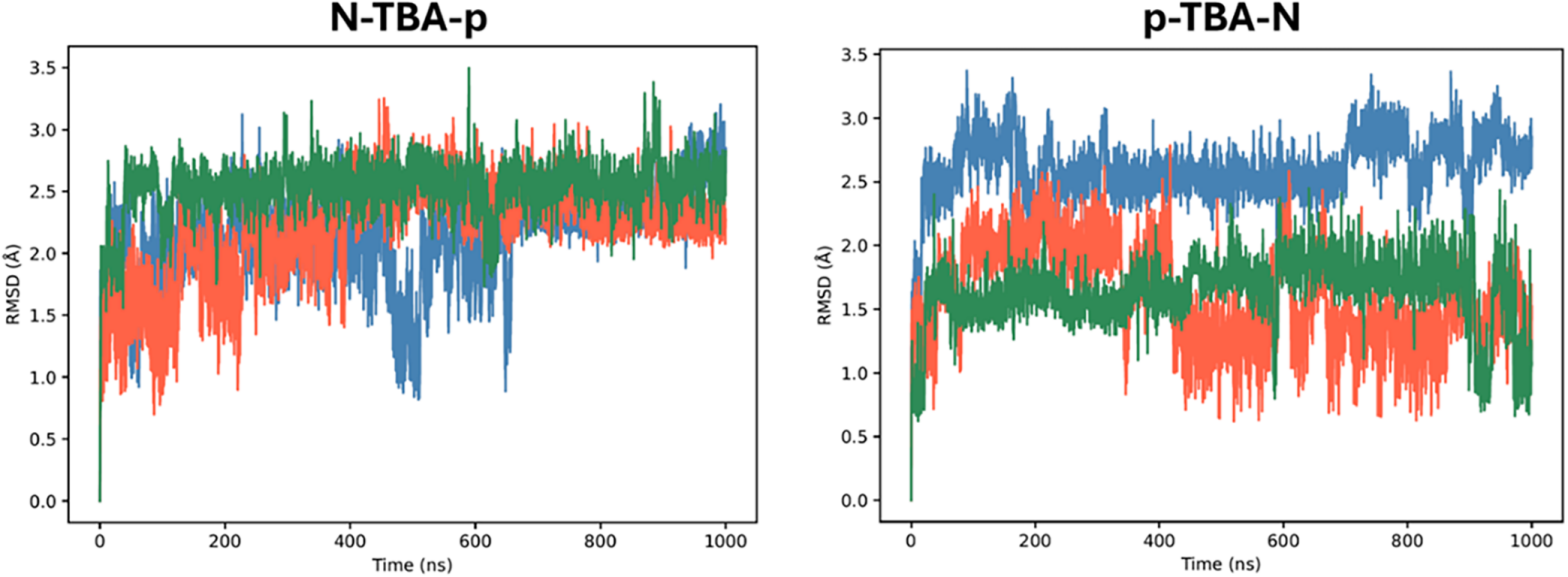
Time-dependent root-mean-square deviation (RMSD) values for replica 1 (blue), replica 2 (orange), and replica 3 (green) of the MD simulations of free **N-TBA-p** and **p-TBA-N**. RMSD values were calculated for all the non-hydrogen atoms and taking as reference the corresponding initial structures after equilibration.

At the end of the MD simulation replicas, the naphthalene diimide of **N-TBA-p** was found to be mainly parallel-stacked on T7 of the G-quadruplex lateral loop (average interaction energy: −3.53 kcal/mol), the terminal −OH of its hydroxypropyl substituent formed a H-bond with O4' of T9 and the hydroxypropyl substituent also formed some hydrophobic interactions with G8 (−2.16 kcal/mol), while the 3-hydroxypropylphosphate pointed toward the solvent (Figures 2 and S2).

On the other hand, in the case of **p-TBA-N**, the naphthalene diimide was mainly offset-stacked on G8 (−4.95 kcal/mol), one of the naphthalene diimide carbonyl oxygen atoms formed a H-bond with the hydrogen of N3 of T7, while the 3-hydroxypropylphosphate pointed to the groove of the G-quadruplex (Figures 3 and S3).

Thus, the presence of fewer and weaker interactions in the case of **p-TBA-N** compared to **N-TBA-p** could justify the highest similarity in melting temperatures between **p-TBA-N** and the parent TBA, as well as the higher thermal stability of **N-TBA-p** compared to **p-TBA-N**.

Moreover, the different chemical interactions of the pendant groups within each aptamer, as well as of the diverse rearrangement of T3, T4, T9, T12, and T13, in the different final 3D structures adopted by the two G-quadruplex-based aptamers (Figure 5), could justify the slightly higher electrophoretic mobility found for **N-TBA-p** than **p-TBA-N**.

**Figure 5:**
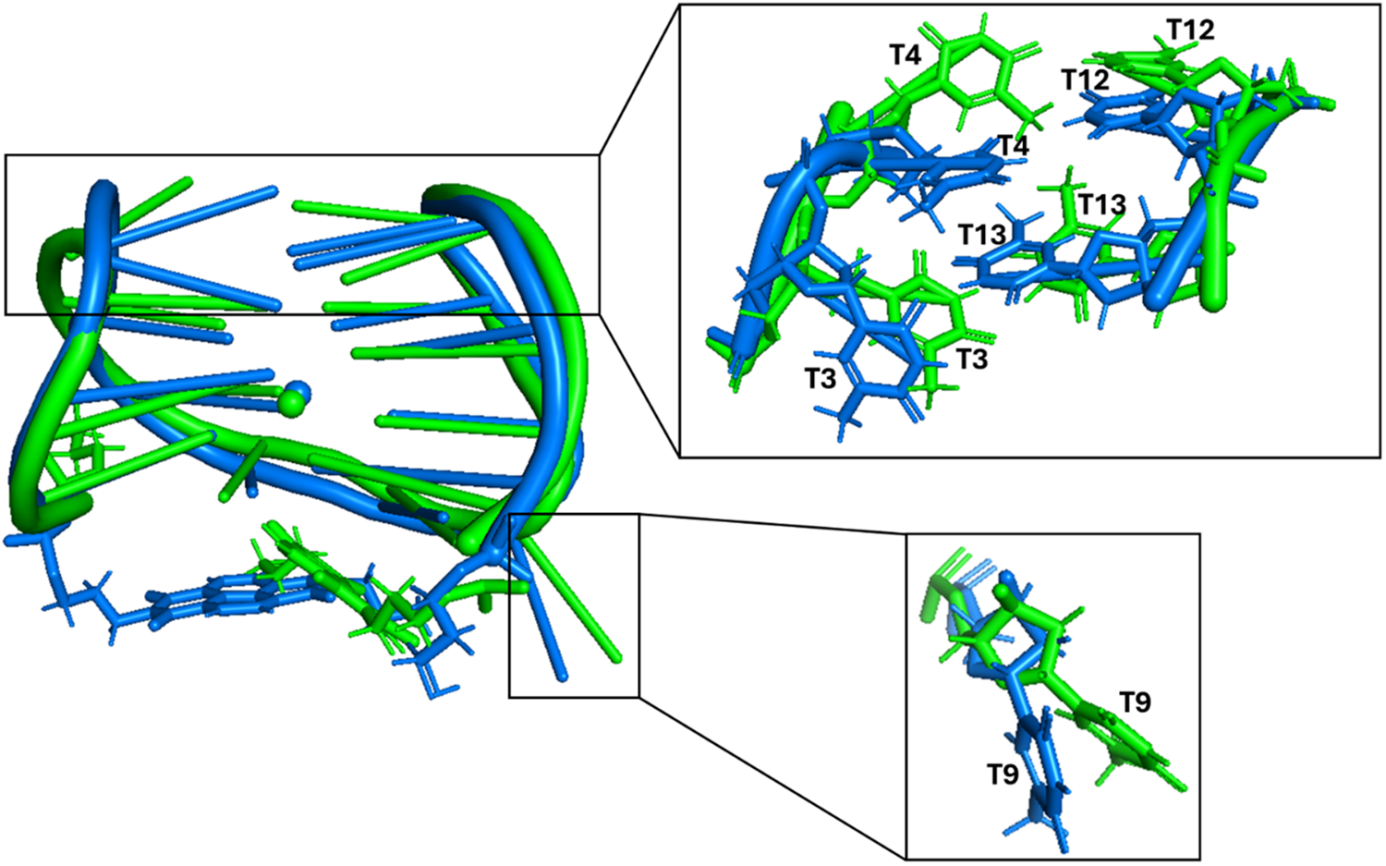
Overlapping of the snapshots from the last frame of the 1 μs MD simulations performed for **N-TBA-p** and **p-TBA-N** (replica 1 of each aptamer). The G-quadruplex aptamers are shown as cartoon, the naphthalene diimide and 3-hydroxypropylphosphate pendant groups as sticks, while Na^+^ ions are represented as spheres. **N-TBA-p** and **p-TBA-N** are colored in blue and green, respectively. Enlargements of the nucleotides with the most different rearrangements between the two aptamers are shown on the right.

Altogether, the stronger stabilizing interactions within **N-TBA-p** as well as the higher compactness of **N-TBA-p** than **p-TBA-N** are in line with the stronger nuclease resistance in serum of **N-TBA-p**, whose 5′- and 3′-ends are more hidden to the degradative enzymes compared to those of **p-TBA-N**.

### 3.2 MD Simulations on Thrombin Interacting with TBA, N-TBA-p, and p-TBA-N

As described above, **N-TBA-p** and **p-TBA-N** showed very different properties also concerning their ability to inhibit the thrombin activity, with **p-TBA-N** having a very similar anticoagulant activity as the parent TBA and **N-TBA-p** showing more than 7 times higher anticoagulant activity.^18^ To explore potential reasons for this different behavior, we evaluated the interactions of **N-TBA-p** and **p-TBA-N** with thrombin by performing MD simulations on their complexes, using the TBA/thrombin complex as reference (Figures 6, 7, and 8). Along each simulation, all of the investigated complexes showed structural stability with an average RMSD of about 1–2 Å, considering either the aptamer or the protein within each complex (Figure 9 and Table S1).

**Figure 6:**
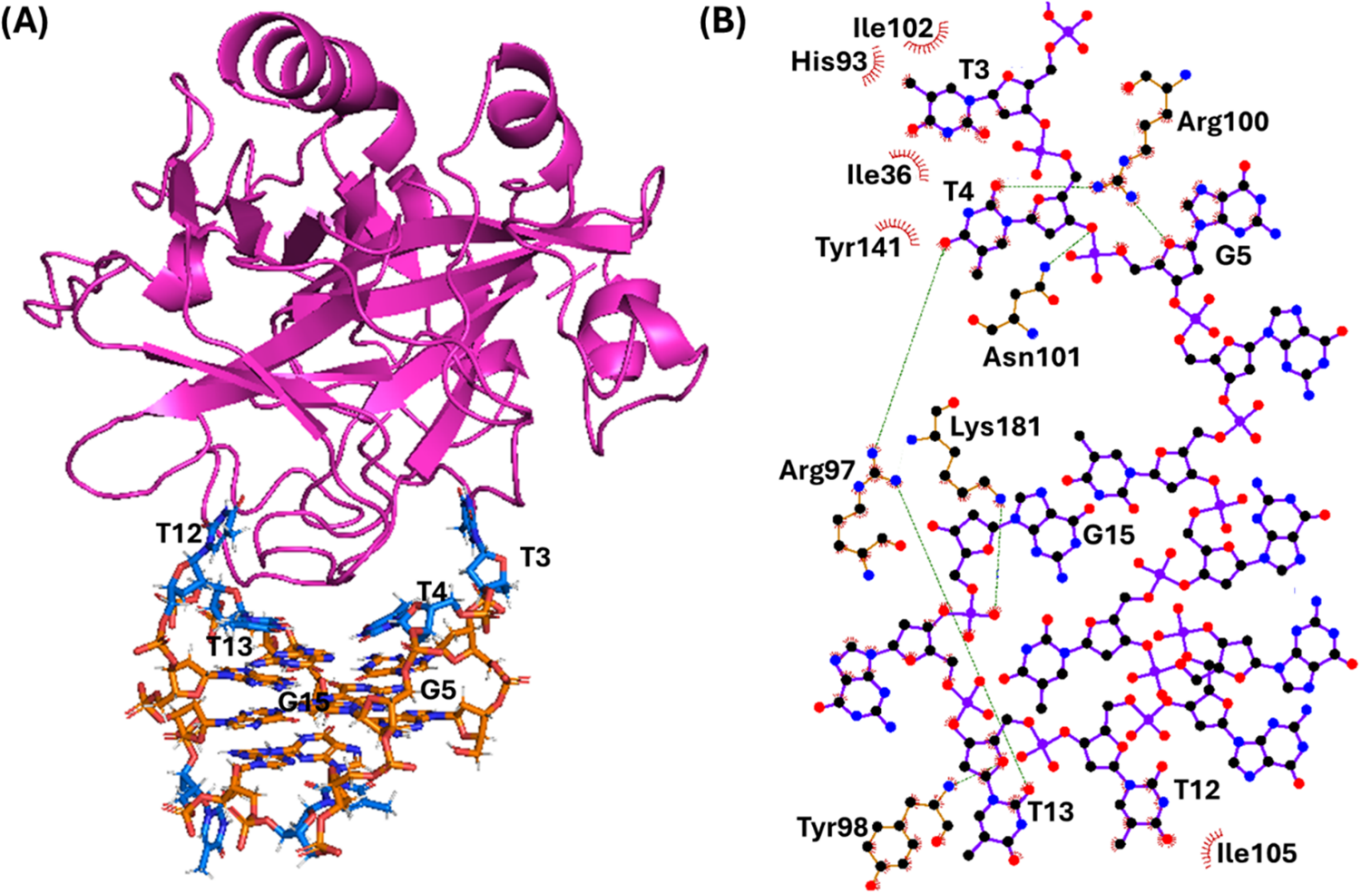
(A) Snapshot from the last frame of the 1 μs MD simulation performed for the TBA/thrombin system (replica 1; for the overlapping of the three replicas, see Figure S4). The G-quadruplex aptamer is shown as a stick, while the protein as a cartoon. Thymidines and guanosines are colored in blue and orange, respectively. Nucleotides involved in the interactions with the protein are labeled. (B) 2D interaction map. C, N, and O atoms are reported in black, blue, and red, respectively. Hydrogen atoms are not depicted for ease of illustration. Hydrogen bonds/electrostatic interactions and hydrophobic contacts are depicted as green dashed lines and red arcs with radiating lines, respectively. Nucleotides, amino acids, and atoms involved in the interactions are labeled.

**Figure 7:**
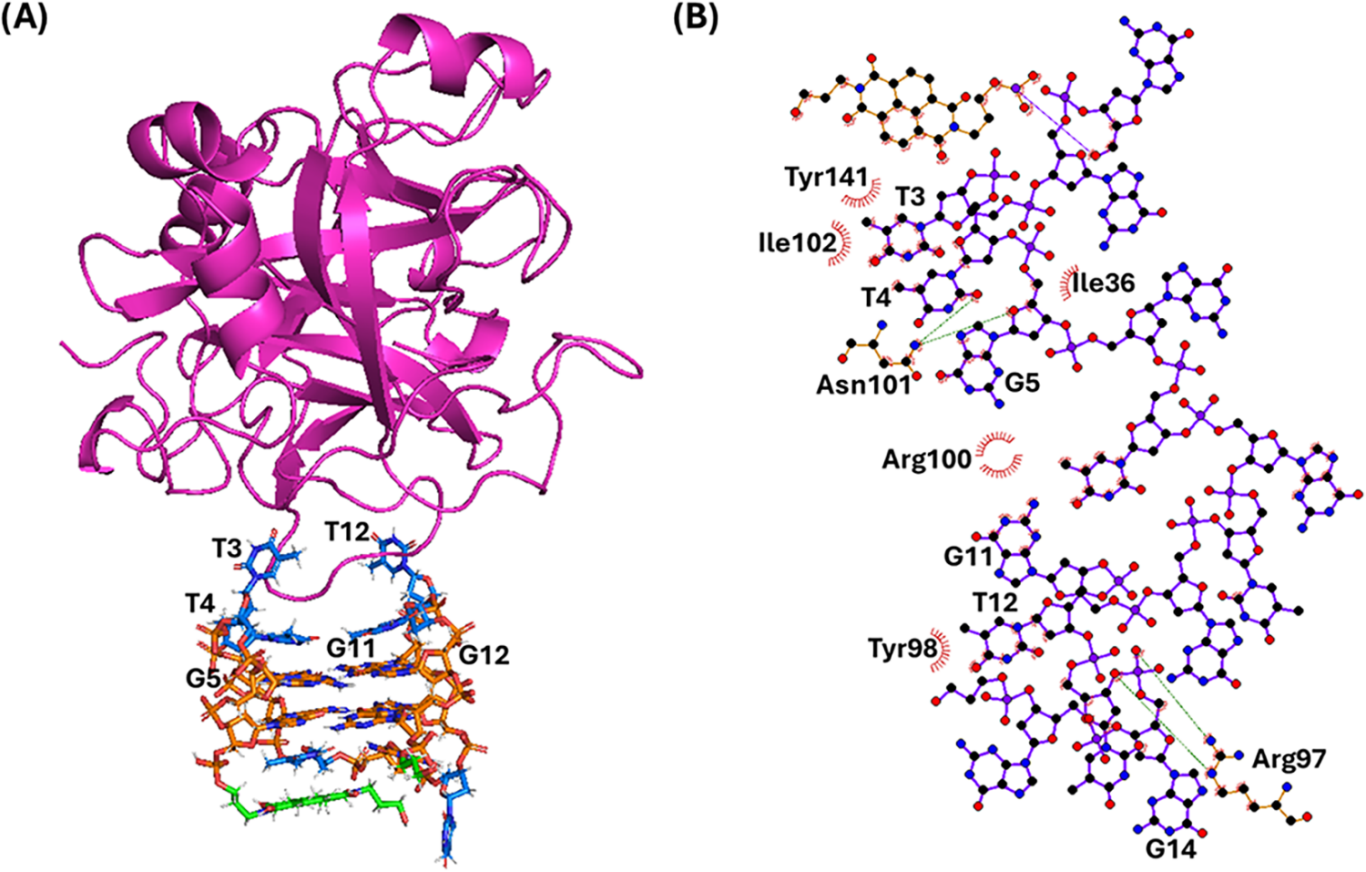
(A) Snapshot from the last frame of the 1 μs MD simulation performed for the **N-TBA-p**/thrombin system (replica 2; for the overlapping of the three replicas, see Figure S5). The G-quadruplex aptamer is shown as a stick, while the protein as a cartoon. Thymidines, guanosines, and the naphthalene diimide and 3-hydroxypropylphosphate pendant groups are colored in blue, orange, and green, respectively. Nucleotides involved in the interactions with the protein are labeled. (B) 2D interaction map. C, N, and O atoms are reported in black, blue, and red, respectively. Hydrogen atoms are not depicted for ease of illustration. Hydrogen bonds/electrostatic interactions and hydrophobic contacts are depicted as green dashed lines and red arcs with radiating lines, respectively. Nucleotides, amino acids, and atoms involved in the interactions are labeled.

**Figure 8:**
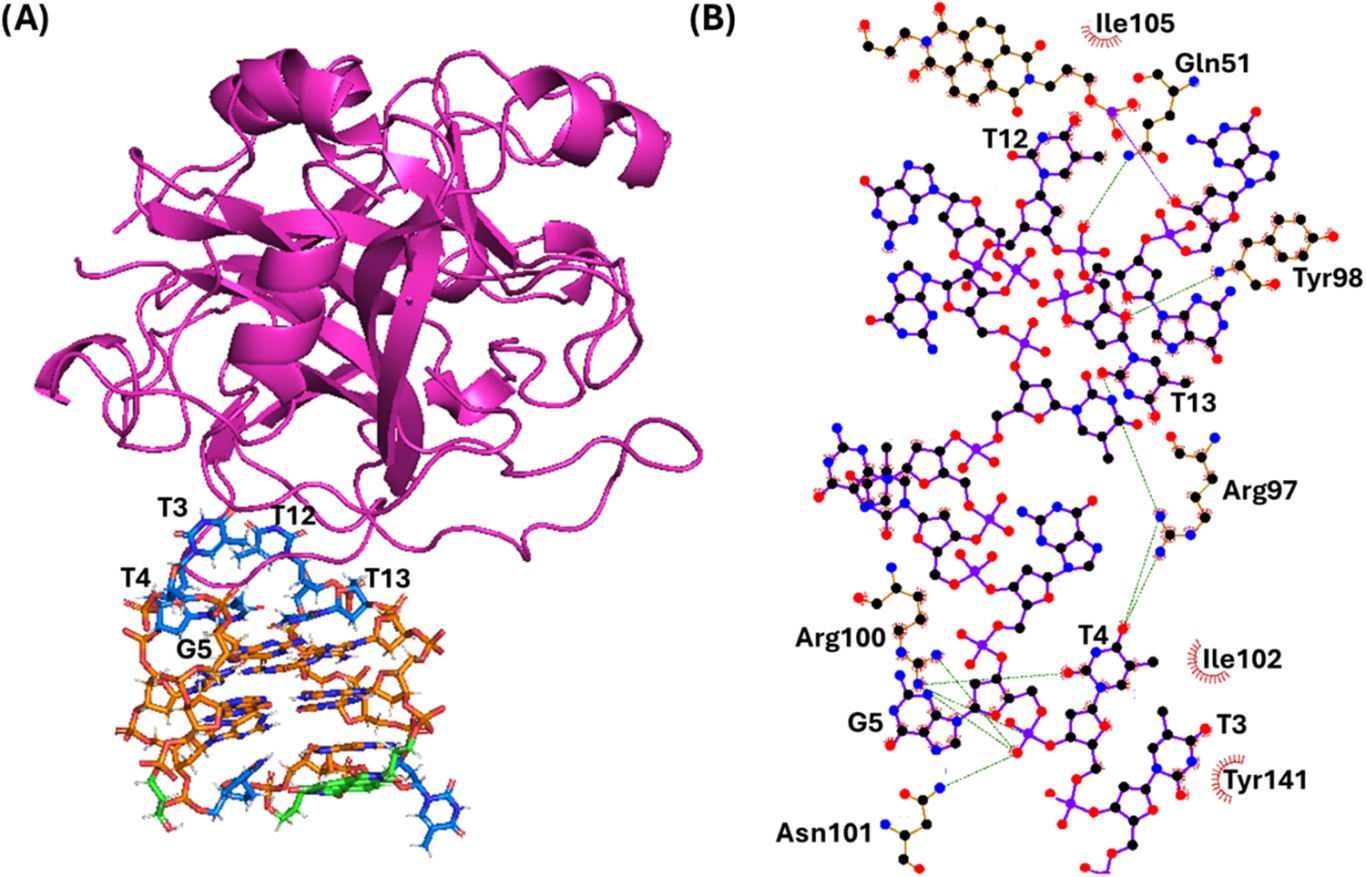
(A) Snapshot from the last frame of the 1 μs MD simulation performed for the **p-TBA-N**/thrombin system (replica 1; for the overlapping of the three replicas, see Figure S6). The G-quadruplex aptamer is shown as a stick, while the protein as a cartoon. Thymidines, guanosines, and the naphthalene diimide and 3-hydroxypropylphosphate pendant groups are colored in blue, orange, and green, respectively. Nucleotides involved in the interactions with the protein are labeled. (B) 2D interaction map. C, N, and O atoms are reported in black, blue, and red, respectively. Hydrogen atoms are not depicted for ease of illustration. Hydrogen bonds/electrostatic interactions and hydrophobic contacts are depicted as green dashed lines and red arcs with radiating lines, respectively. Nucleotides, amino acids, and atoms involved in the interactions are labeled.

**Figure 9:**
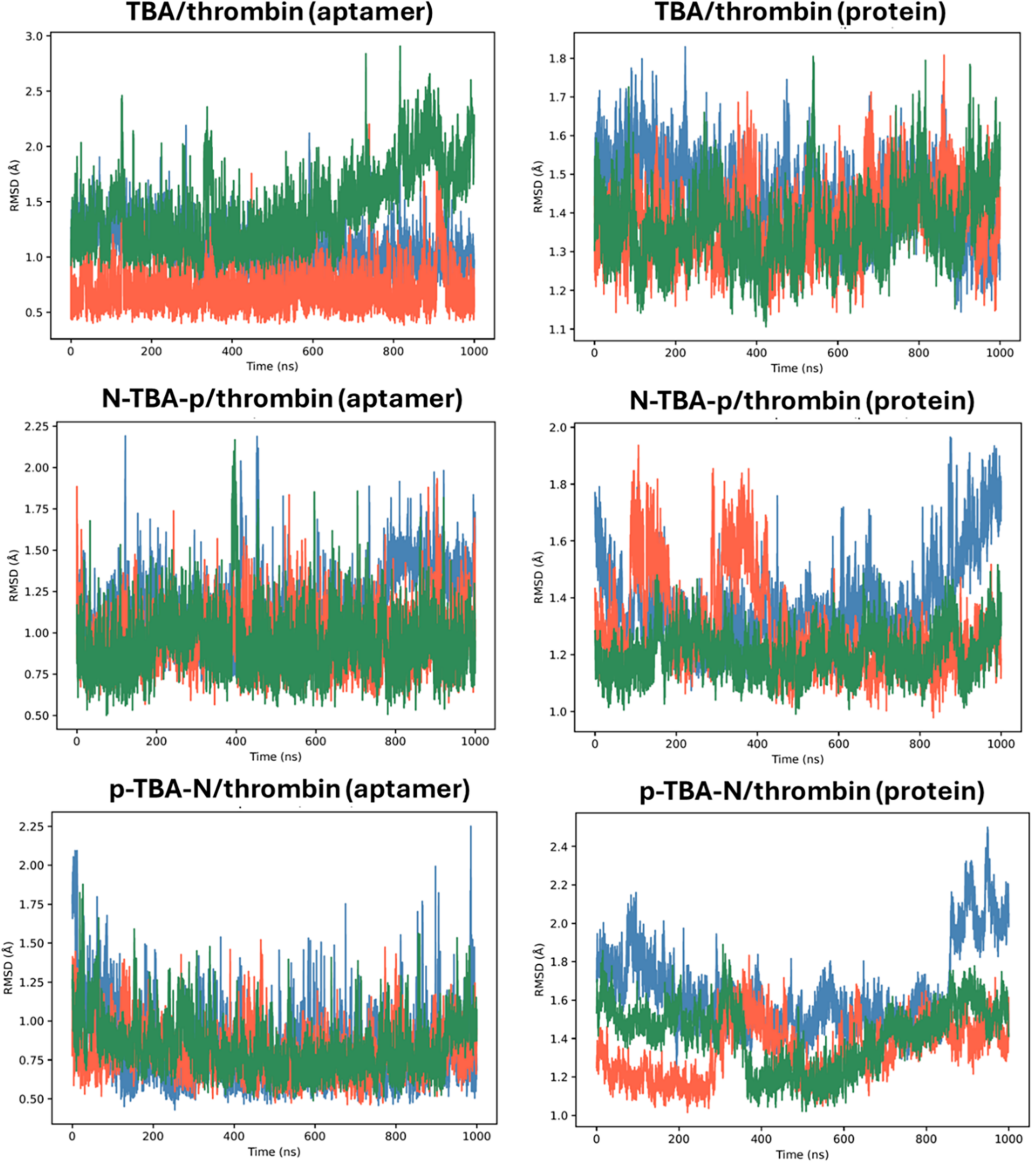
Time-dependent root-mean-square deviation (RMSD) values for replica 1 (blue), replica 2 (orange), and replica 3 (green) of the MD simulations of the systems TBA/thrombin, **N-TBA-p**/thrombin, and **p-TBA-N**/thrombin. RMSD values were calculated for all the non-hydrogen atoms, both for the aptamer and protein, and taking as reference the corresponding initial structures after equilibration.

In the case of TBA/thrombin system, the following main interactions between the aptamer and protein were found (Figures 6 and S4): 1) hydrogen bonds between O2 of T4, O4' of G5, and the guanidium group of Arg100, between O4 of T4, and O2 of T13, and the guanidium group of Arg97, as well as between O3' of T4 and NH_2_ of Asn101; 2) electrostatic interaction between the phosphate group of G15 and the protonated amino group of Lys181; 3) T-shaped stacking between T3 and Tyr141 and stacking interaction between T12 and Tyr98; 4) hydrophobic interactions between T3 and Ile36, His93, and Ile102 and between T12 and Ile105.

On the other hand, for **N-TBA-p**/thrombin system, the naphthalene diimide moiety of **N-TBA-p** was stably stacked on T7 of the G-quadruplex lateral loop, and the 3-hydroxypropylphosphate pointed toward the solvent as in the free **N-TBA-p** aptamer (Figures 7 and S5). Additionally, the following main interactions were found at the interface between the aptamer and protein (Figure 7): 1) hydrogen bonds between O2 of T4, O4' of G5, and the NH_2_ group of Asn101; 2) electrostatic interaction between the phosphate group of G14 and the guanidium group of Arg97; 3) T-shaped stacking between T3 and Tyr141 and stacking interaction between T12 and Tyr98; 4) hydrophobic interactions between G5, G11, and Arg100, between T3 and Ile36, and between T3, T4, and Ile102.

Finally, for **p-TBA-N**/thrombin system, the naphthalene diimide was only partially stacked on G8 of the G-quadruplex lateral loop and the 3-hydroxypropylphosphate can point toward either the G-quadruplex groove or the solvent differently from the free **p-TBA-N** aptamer (Figures 8 and S6). In addition, the following main interactions were found at the interface between the aptamer and protein (Figure 8): 1) hydrogen bonds between the phosphate group of T13 and NH_2_ of Gln51, between the O4̀' of T13 and NH of Tyr98, between the O2 of T13 and the guanidium group of Arg97, between the O2 of T4 and the guanidium group of Arg100, and between the phosphate group of G5 and NH_2_ of Asn101; 2) T-shaped stacking between T3 and Tyr141 and stacking interaction between T12 and Tyr98; and 3) hydrophobic interactions between T3 and Ile102 and between T12 and Ile105.

Notably, a higher flexibility of **N-TBA-p** compared to that of **p-TBA-N** was observed in their complexes with thrombin (Figures S5 and S6), as also confirmed by the inspection of RMSF values, which showed the higher fluctuations of both G-quadruplex and pendant groups in **N-TBA-p** than **p-TBA-N** (Figure 10). On the other hand, similar RMSF values were found for thrombin in both complexes, denoting the similar, stable conformation adopted by the protein in the two systems (Figure 10).

**Figure 10:**
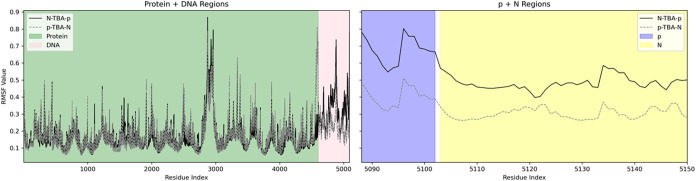
Root-mean-square fluctuation (RMSF) values (Å) for **N-TBA-p**/thrombin (solid line) and **p-TBA-N**/thrombin (dotted line) systems. Protein, DNA, **p**, and **N** residues are highlighted by green, pink, purple, and yellow panels, respectively.

Moreover, upon detailed comparison of the different MD replicas for TBA/thrombin, **N-TBA-p**/thrombin, and **p-TBA-N**/thrombin systems, while the antiparallel G-quadruplex portion of the aptamers as well as the main G-quadruplex/protein interface contacts proved to be preserved comparing TBA, **N-TBA-p**, and **p-TBA-N**, significant structural differences were observed in the protein region in close proximity to the thrombin exosite I,^40^ involving the amino acids Asn178, Val179, Gly180, and Lys181 (Figure 11). Indeed, only in the case of **N-TBA-p**/thrombin system, this region can be directed toward and interacted with the aptamer, as observed in replica 1, thus forming the following additional bonds: (1) hydrogen bonds between O3' of G14 and NH of Lys181 as well as between the phosphate group of G15 and NH_2_ of Asn178; (2) electrostatic interaction between the phosphate group of G14 and the protonated amino group of Lys181; and (3) hydrophobic interactions between T13, G14, G15 and Val179, Gly180.

**Figure 11:**
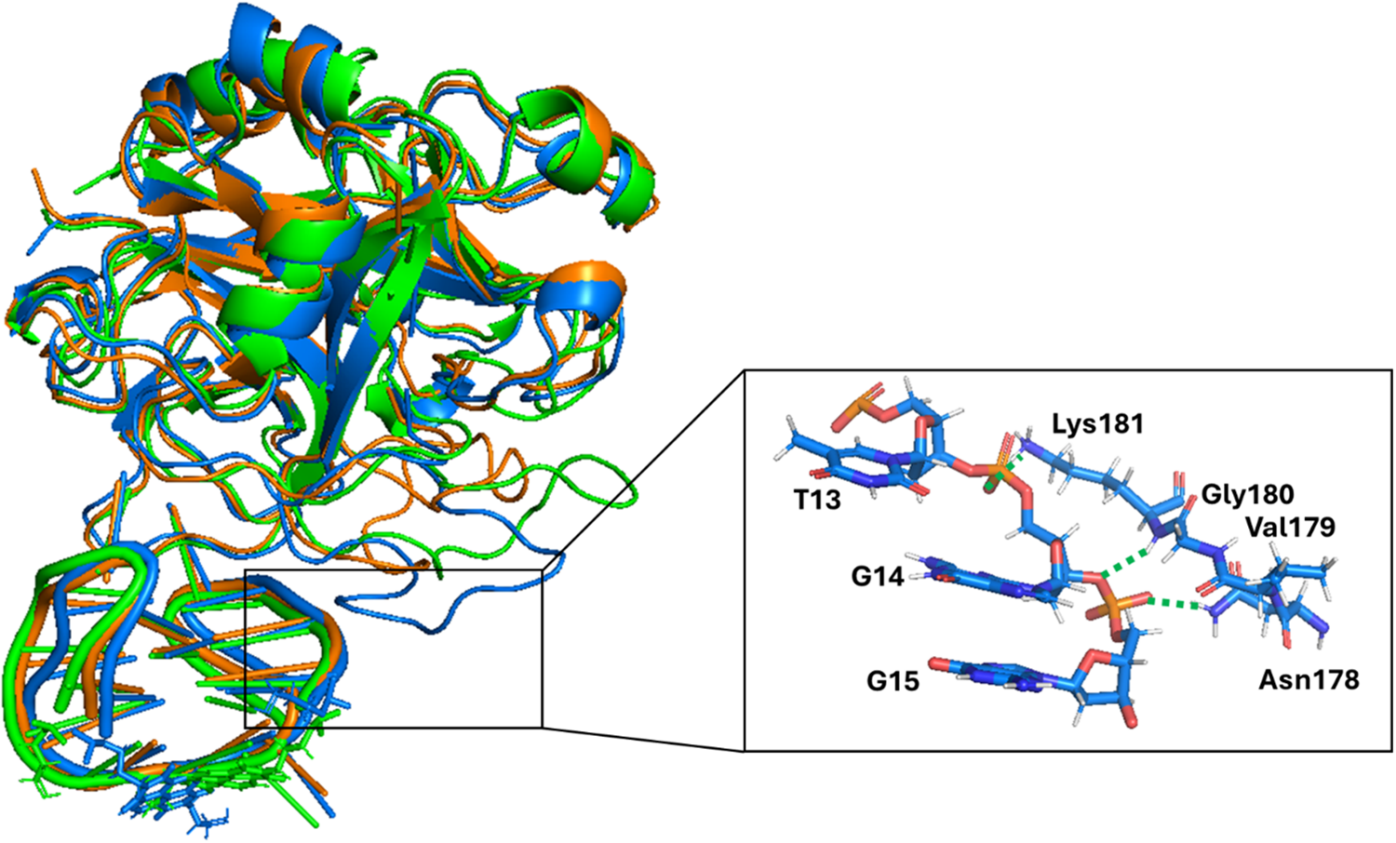
Overlapping of the snapshots from the last frame of the 1 μs MD simulations performed for TBA/thrombin, **N-TBA-p**/thrombin, and **p-TBA-N**/thrombin systems (replica 1 of each system). The G-quadruplex aptamers and protein are shown as a cartoon, while the naphthalene diimide and 3-hydroxypropylphosphate pendant groups as sticks. TBA/thrombin, **N-TBA-p**/thrombin, and **p-TBA-N**/thrombin systems are colored in orange, blue, and green, respectively. Enlargement of the protein region with the most different rearrangements between the three systems is shown on the right, and the interactions between **N-TBA-p** and thrombin are highlighted. Hydrogen bonds/electrostatic interactions are depicted as green dashed lines. Nucleotides and amino acids involved in the interactions are labeled.

Noteworthy is the role of the 3-hydroxypropylphosphate pendant group in determining the difference in the interaction with thrombin of **N-TBA-p** compared to **p-TBA-N** and TBA. Indeed, it seems that the 3-hydroxypropylphosphate moiety is the first responsible for the recruitment of the protein region involving the amino acids Asn178, Val179, Gly180, and Lys181, observed in the time frame 830–990 ns (Figure 12), successively allowing these amino acid residues to eventually get close to T13, G14, G15 of **N-TBA-p** G-quadruplex as observed after 1 μs MD simulation (Figure 11). Notably, by calculating the distance between Val179 and the 3-hydroxypropylphosphate group throughout each MD simulation (Figure 13A), as well as the distances between the interacting atoms of aptamer and protein (Figure 13B) shown in Figure 11, it is clear that the 3-hydroxypropylphosphate group gets in close contact with the protein during all three MD simulation replicas performed for **N-TBA-p**/thrombin system and then G14 and G15 move toward Lys181 and Asn178, respectively, forming the found hydrogen bonds and electrostatic interactions. This behavior is only possible for **N-TBA-p** having the highly floating 3-hydroxypropylphosphate in close proximity to the highly flexible protein region involved in the binding. On the other hand, in the case of **p-TBA-N**, the naphthalene diimide pendant group is on the same side of the flexible protein region and, due to its more stable interactions with the G-quadruplex aptamer core, is unable to interact with any region of the protein. Indeed, a higher number of hydrogen bonds showing a higher stability over the MD simulation time was observed between thrombin and the 3-hydroxypropylphosphate group as well as between the aptamer and the naphthalene diimide unit for **N-TBA-p** compared to **p-TBA-N** (Figure S7), in agreement with the differential interactions observed between the two aptamers. Moreover, a higher number of hydrogen bonds was observed between the aptamer and the 3-hydroxypropylphosphate group for **p-TBA-N** compared to **N-TBA-p** so that the 3-hydroxypropylphosphate pendant group (Figure S7) results generally more hidden to potential interactions with the protein in the case of **p-TBA-N**. Finally, a similar number of hydrogen bonds was found both intra-aptamer and at the G-quadruplex/protein interface (Figure S7) in agreement with the retention of the antiparallel G-quadruplex portion as well as the main G-quadruplex/protein interface contacts both in the case of **N-TBA-p** and **p-TBA-N**.

**Figure 12:**
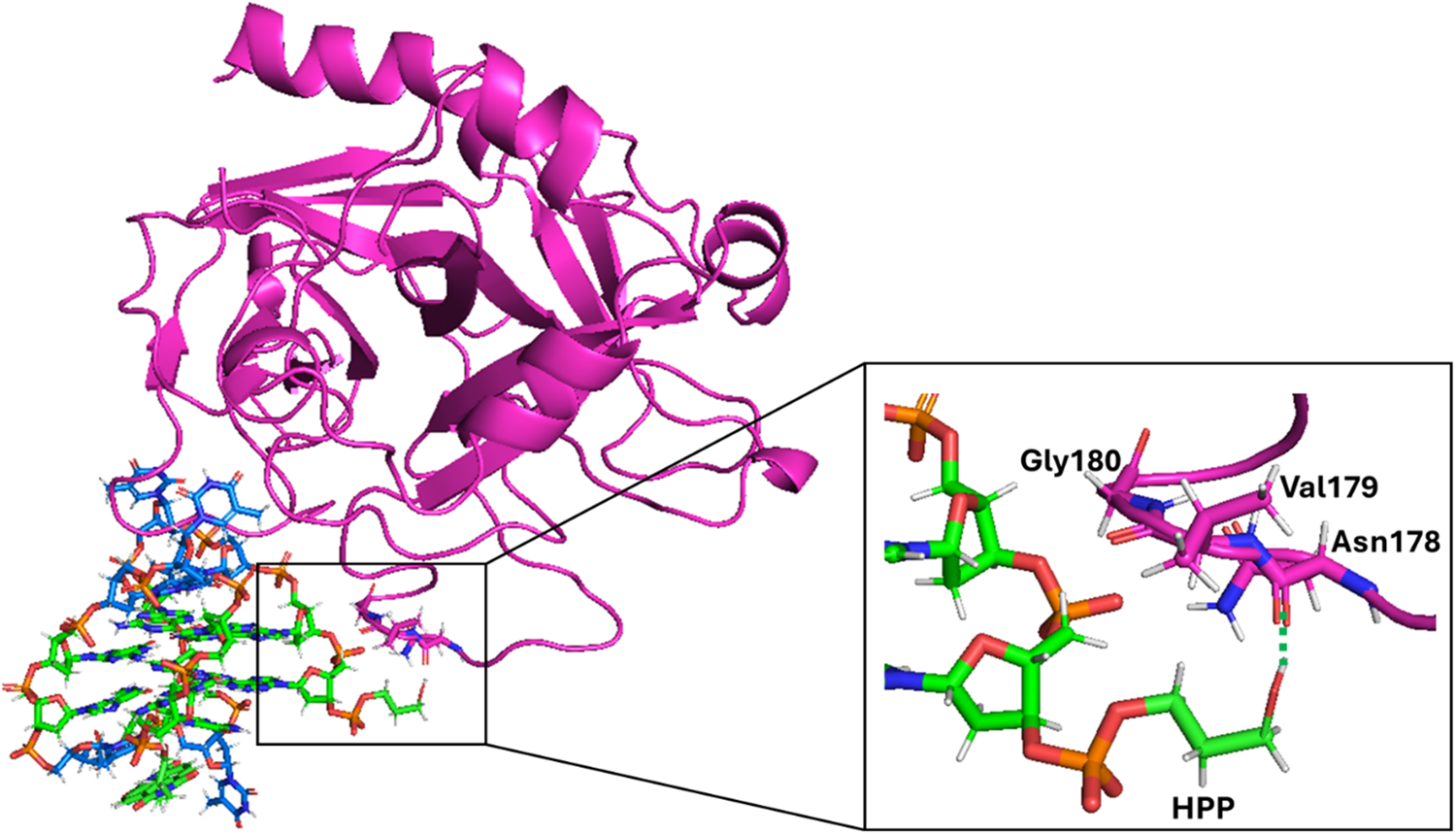
Snapshot from the frame at 990 ns of the MD simulation performed for the **N-TBA-p**/thrombin system (replica 1). The G-quadruplex aptamer is shown as a stick, while the protein and the amino acids involved in interactions as cartoons and sticks, respectively. Thymidines, guanosines, and the naphthalene diimide and 3-hydroxypropylphosphate pendant groups are colored in blue, orange, and green, respectively. Enlargement of the interactions between **N-TBA-p** and thrombin is shown on the right. The hydrogen bond is depicted as green dashed lines. Amino acids involved in the interactions with the 3-hydroxypropylphosphate (HPP) pendant group are labeled.

**Figure 13:**
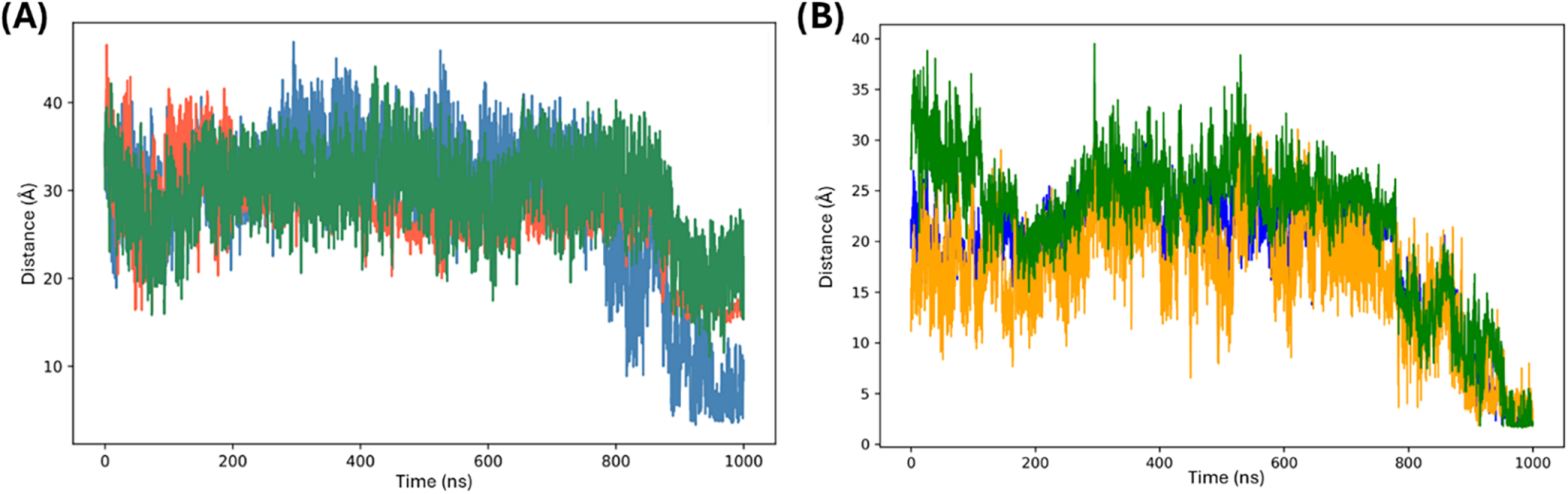
(A) Distance between Val179 and 3-hydroxypropylphosphate group for replica 1 (blue), replica 2 (orange), and replica 3 (green) of the MD simulations of **N-TBA-p**/thrombin system. (B) Distance between O3' of G14 and NH of Lys181 (blue), between the phosphate group of G14 and the protonated amino group of Lys181 (yellow), and between the phosphate group of G15 and NH_2_ of Asn178 (green) for replica 1 of the MD simulations of **N-TBA-p**/thrombin system.

Overall, the additional interactions with the protein observed for **N-TBA-p** compared to **p-TBA-N** resulted in a higher interaction energy between aptamer and protein for the **N-TBA-p**/thrombin complex (average interaction energy: −120.8 kcal/mol) compared to the **p-TBA-N**/thrombin (−99.6 kcal/mol) complex. Thus, the stronger affinity found for **N-TBA-p** toward thrombin can finally explain the higher anticoagulant activity found for **N-TBA-p** compared to **p-TBA-N**.

## 4 Conclusions

Searching for novel and more efficient anticoagulant agents, the thrombin-binding aptamer TBA, forming an antiparallel G-quadruplex structure, was in-depth investigated during the last decades, particularly developing a plethora of different modified analogues.^14^ Aiming at improving its thermal stability, nuclease resistance in serum, and anticoagulant activity, some of us recently designed and synthesized several analogues of TBA bearing a naphthalene diimide and/or 3-hydroxypropylphosphate moieties either at the 5′- or 3′-end.^18^ Among them, **N-TBA-p** emerged as the best analogue in the explored series. It exhibited remarkably improved properties compared to unmodified TBA, proving to be very resistant in serum and extremely effective as a thrombin inhibitor and thus representing a promising candidate for in vivo studies. On the other hand, **p-TBA-N**, having the same pendant groups at 5′- and 3′-end as **N-TBA-p** but in reversed positions, was one of the least investigated analogues, both in terms of nuclease resistance and thrombin inhibition.^18^ These experimental results clearly proved that the same pendant groups at different ends of the TBA G-quadruplex structure can dramatically affect the properties of thrombin-binding aptamers, which stimulated us to investigate the structural reasons behind the observed peculiar features for the newly designed TBA analogues.

Thus, aiming at elucidating the role of the naphthalene diimide and 3-hydroxypropylphosphate pendant groups in determining the different behavior between **N-TBA-p** and **p-TBA-N**, also in comparison with the parent TBA, MD simulations on free aptamers as well as on the aptamers interacting with thrombin were performed.

From our careful analysis, it emerged that the naphthalene diimide pendant group is involved in a higher number and stronger interactions with the G-quadruplex core in the case of **N-TBA-p** compared to **p-TBA-N** determining the higher thermal stability of **N-TBA-p** than **p-TBA-N** and TBA. Moreover, a diverse rearrangement of T3, T4, T9, T12, and T13 is observed in the two G-quadruplex-based aptamers, resulting in higher compactness of **N-TBA-p** than **p-TBA-N**. Altogether, the stronger stabilizing interactions within **N-TBA-p** as well as the higher compactness of **N-TBA-p** than **p-TBA-N** can explain the stronger nuclease resistance in serum of **N-TBA-p**, whose 5′- and 3′-ends result more hidden to the degradative enzymes compared to those of **p-TBA-N**.

As far as the interaction with thrombin is concerned, significant structural differences were observed in the protein region in close proximity to the thrombin exosite I, involving the amino acids Asn178, Val179, Gly180, and Lys181, upon comparison of TBA/thrombin, **N-TBA-p**/thrombin, and **p-TBA-N**/thrombin systems. Particularly, the 3-hydroxypropylphosphate pendant group proved to be crucial in determining the difference in the interaction with thrombin of **N-TBA-p** compared to **p-TBA-N** and TBA. Indeed, when in proximity to the highly flexible protein region close to exosite I, i.e., only in the case of **N-TBA-p**, the highly floating 3-hydroxypropylphosphate is able to recruit the protein region involving the amino acids Asn178, Val179, Gly180, and Lys181, successively allowing these amino acid residues to get close to T13, G14, and G15 of the **N-TBA-p** G-quadruplex which can form additional interactions with the protein, in turn missing in the case of **p-TBA-N** and TBA. These additional interactions result in the stronger affinity of **N-TBA-p** to thrombin and can explain the higher anticoagulant activity found for **N-TBA-p** than **p-TBA-N**.

Notably, the insights found here for **N-TBA-p** and **p-TBA-N**, explaining their different properties as thrombin aptamers, can also be extended to the other aptamers of the same series.^18^ Indeed, among them, the aptamers bearing the 3-hydroxypropylphosphate at the G-quadruplex 3′-end, such as **TBA-Np**, **p-TBA-Np**, and **p-TBA-p**, were more effective in terms of anticoagulant activity compared to the ones without the 3-hydroxypropylphosphate at the 3′-end. Moreover, our findings might be, in principle, extended to other aptamers targeting thrombin, specifically preserving the antiparallel G-quadruplex core of the parent TBA aptamer.

Overall, our MD studies provided the rationale behind the different behavior of **N-TBA-p** and **p-TBA-N** and highlighted the importance of linking more rigid pendant groups, able to form stabilizing interactions with the antiparallel TBA G-quadruplex, at the G-quadruplex 5′-end as well as highly flexible pendant groups, carrying H-bond donors/acceptors, at the G-quadruplex 3′-end, which is crucial in view of rationally designing and obtaining systems better interacting with thrombin, thus potentially producing a more potent protein inhibition.

## Supplementary Material



## Data Availability

**N-TBA-p**: https://mdposit.mddbr.eu/#/id/MD-A003ZU/overview. **p-TBA-N**: https://mdposit.mddbr.eu/#/id/MD-A003ZV/overview. TBA/thrombin: https://mdposit.mddbr.eu/#/id/MD-A003ZW/overview. **N-TBA-p**/thrombin: https://mdposit.mddbr.eu/#/id/MD-A003ZX/overview. **p-TBA-N**/thrombin: https://mdposit.mddbr.eu/#/id/MD-A003ZY/overview.

## Notes

Chemical structures of TBA analogues; RMSD values for the MD simulations; overlapping of the MD simulations replicas; and total number of hydrogen bonds (PDF)
