# Supplementary material for: When the Position of Pendant Groups Makes the Difference in G‑Quadruplex Behavior: The Case of Bis-Conjugated Thrombin-Binding Aptamers
Source: J Chem Inf Model. 2025 Dec 24;66(1):642–52. doi: 10.1021/acs.jcim.5c01598 (PMC12801291; doi:10.1021/acs.jcim.5c01598)
Supplement: Supplementary file 1 [file ci5c01598_si_001.pdf]

Supporting Information

When the Position of Pendant Groups Makes the Difference in G-Quadruplex Behaviour: the Case of Bis-Conjugated Thrombin Binding Aptamers

Chiara Platella,[a,*] Federica Battistini,[b,c] Claudia Riccardi,[a] Michael Smietana,[d] François Morvan,[d] Modesto Orozco,[b,c,*] Daniela Montesarchio[a]

[a]*Department of Chemical Sciences, University of Naples Federico II, 80126 Naples, Italy*

[b]*Institute for Research in Biomedicine (IRB Barcelona), The Barcelona Institute of Science and Technology, 08028 Barcelona, Spain*

[c]*Department of Biochemistry and Biomedicine, University of Barcelona, 08028 Barcelona, Spain*

[d]*Institut des Biomolécules Max Mousseron, Université de Montpellier, CNRS, ENSCM, 34293 Montpellier, France*

**Corresponding authors: Chiara Platella (chiara.platella@unina.it); Modesto Orozco (modesto.orozco@irbbarcelona.org)*

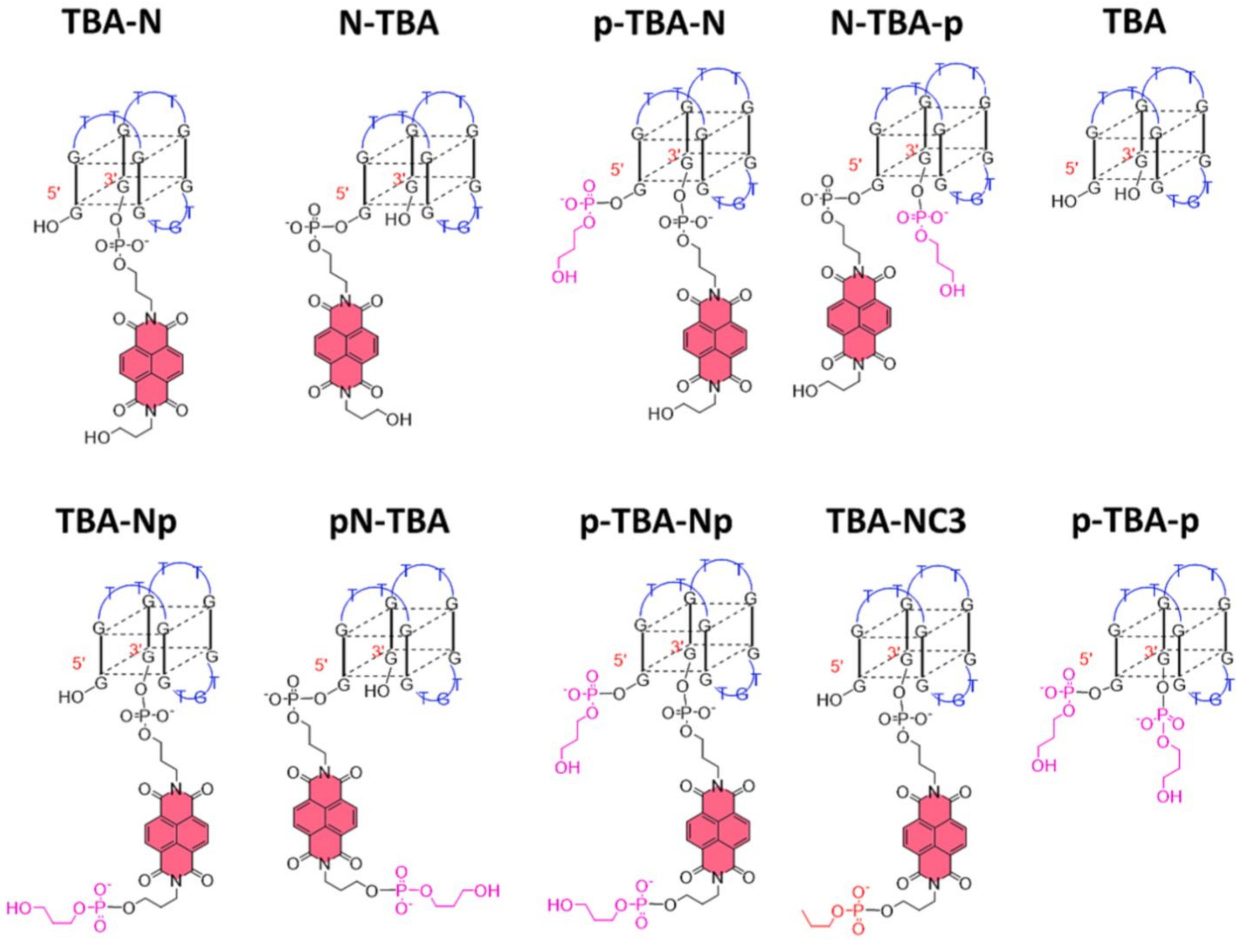

Figure S1. Chemical structures of all TBA analogues designed and synthesized in our previous study. **N** = naphthalene diimide; **p** = 3-hydroxypropylphosphate; C3 = *n*-propyl phosphate. Reproduced with permission from ref [1]. Copyright 2023 Elsevier.

[1] C. Riccardi, K. Pérez de Carvasal, C. Platella, A. Meyer, M. Smietana, F. Morvan, D. Montesarchio, Probing naphthalene diimide and 3-hydroxypropylphosphate as end-conjugating moieties for improved thrombin binding aptamers: structural and biological effects, Bioorg. Chem. 141 (2023) 106917. https://doi.org/10.1016/j.bioorg.2023.106917.

Table S1. Average RMSD values for the three replica copies of the MD simulations of free **N-TBA-p** and **p-TBA-N** and the TBA/thrombin, **N-TBA-p**/thrombin and **p-TBA-N**/thrombin systems. Average RMSD values were calculated for all the non-hydrogen atoms and taking as reference the corresponding initial structures after equilibration. For TBA/thrombin, **N-TBA-p**/thrombin and **p-TBA-N**/thrombin systems, RMSD values were calculated both for the aptamer and protein.

	**RMSD (±SD) (Å)**		
	Replica 1	Replica 2	Replica 3
**N-TBA-p**	2.13 (±0.36)	2.19 (±0.41)	2.53 (±0.22)
**p-TBA-N**	2.61 (±0.25)	1.61 (±0.37)	1.63 (±0.27)
**TBA/thrombin**	1.62 (±0.36) (aptamer)<br>2.00 (±0.18) (protein)	1.22 (±0.24) (aptamer)<br>1.99 (±0.20) (protein)	1.58 (±0.14) (aptamer)<br>1.89 (±0.14) (protein)
**N-TBA-p/thrombin**	1.38 (±0.27) (aptamer)<br>2.23 (±0.24) (protein)	1.27 (±0.19) (aptamer)<br>1.93 (±0.15) (protein)	1.34 (±0.24) (aptamer)<br>1.75 (±0.13) (protein)
**p-TBA-N/thrombin**	1.96 (±0.16) (aptamer)<br>2.35 (±0.28) (protein)	1.09 (±0.13) (aptamer)<br>1.82 (±0.25) (protein)	1.41 (±0.18) (aptamer)<br>2.07 (±0.34) (protein)

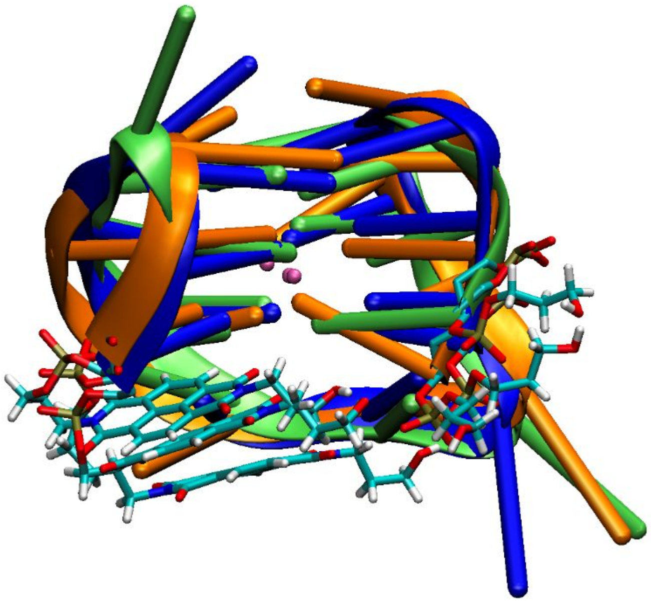

Figure S2. Overlapping of the snapshots from the last frame of replica 1 (blue), replica 2 (orange) and replica 3 (green) of the 1 μs MD simulations performed for **N-TBA-p**. The G-quadruplex aptamers are shown as cartoon, the naphthalene diimide and 3-hydroxypropylphosphate pendant groups as sticks, while Na^+ ions are represented as spheres.

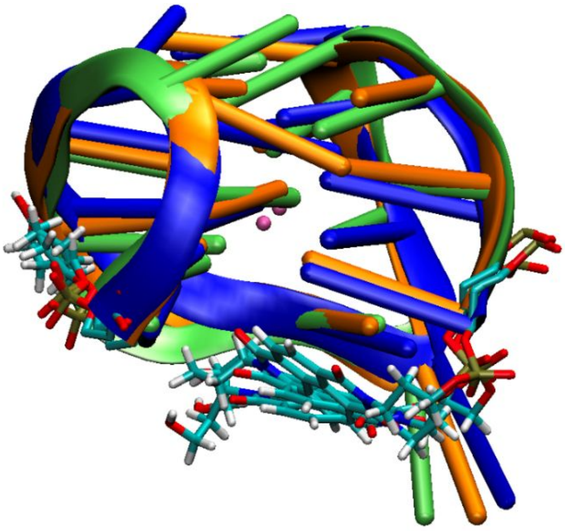

Figure S3. Overlapping of the snapshots from the last frame of replica 1 (blue), replica 2 (orange) and replica 3 (green) of the 1 μs MD simulations performed for **p-TBA-N**. The G-quadruplex aptamers are shown as cartoon, the naphthalene diimide and 3-hydroxypropylphosphate pendant groups as sticks, while Na^+ ions are represented as spheres.

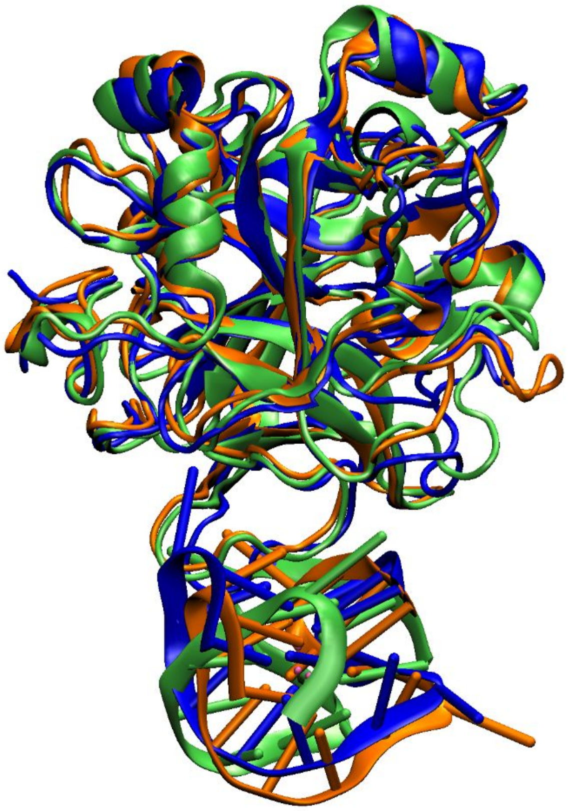

Figure S4. Overlapping of the snapshots from the last frame of replica 1 (blue), replica 2 (orange) and replica 3 (green) of the 1 μs MD simulations performed for TBA/thrombin. The G-quadruplex aptamers are shown as cartoon, the naphthalene diimide and 3-hydroxypropylphosphate pendant groups as sticks, while Na^+ ions are represented as spheres.

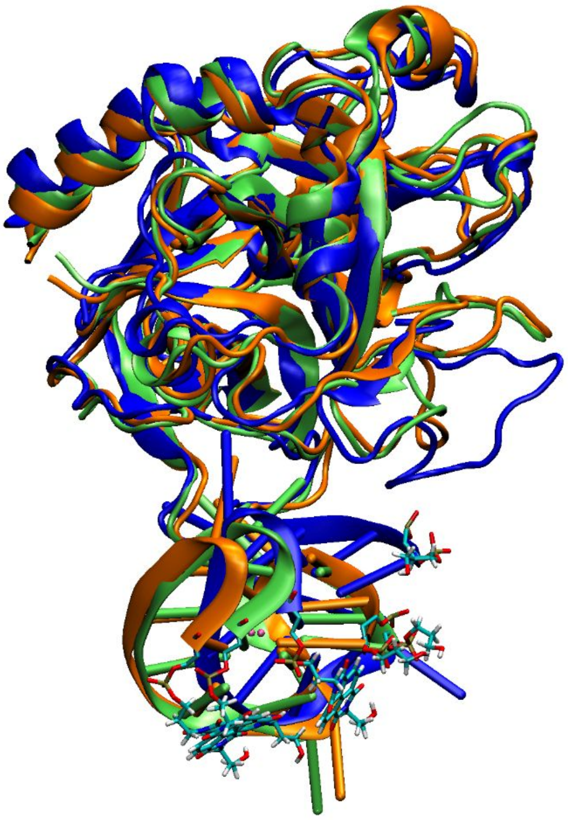

Figure S5. Overlapping of the snapshots from the last frame of replica 1 (blue), replica 2 (orange) and replica 3 (green) of the 1 μs MD simulations performed for **N-TBA-p**/thrombin. The G-quadruplex aptamers are shown as cartoon, the naphthalene diimide and 3-hydroxypropylphosphate pendant groups as sticks, while Na^{+} ions are represented as spheres.

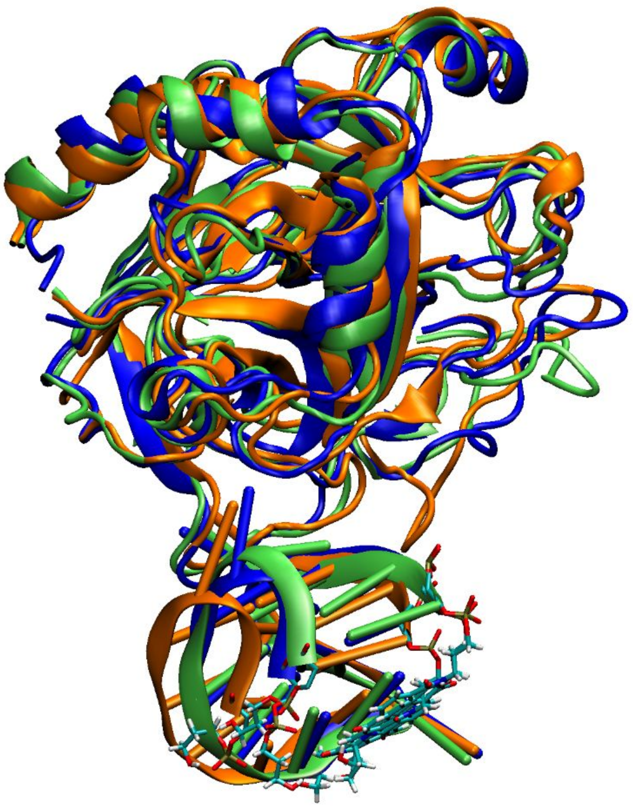

Figure S6. Overlapping of the snapshots from the last frame of replica 1 (blue), replica 2 (orange) and replica 3 (green) of the 1 μs MD simulations performed for **p-TBA-N**/thrombin. The G-quadruplex aptamers are shown as cartoon, the naphthalene diimide and 3-hydroxypropylphosphate pendant groups as sticks, while Na^+ ions are represented as spheres.

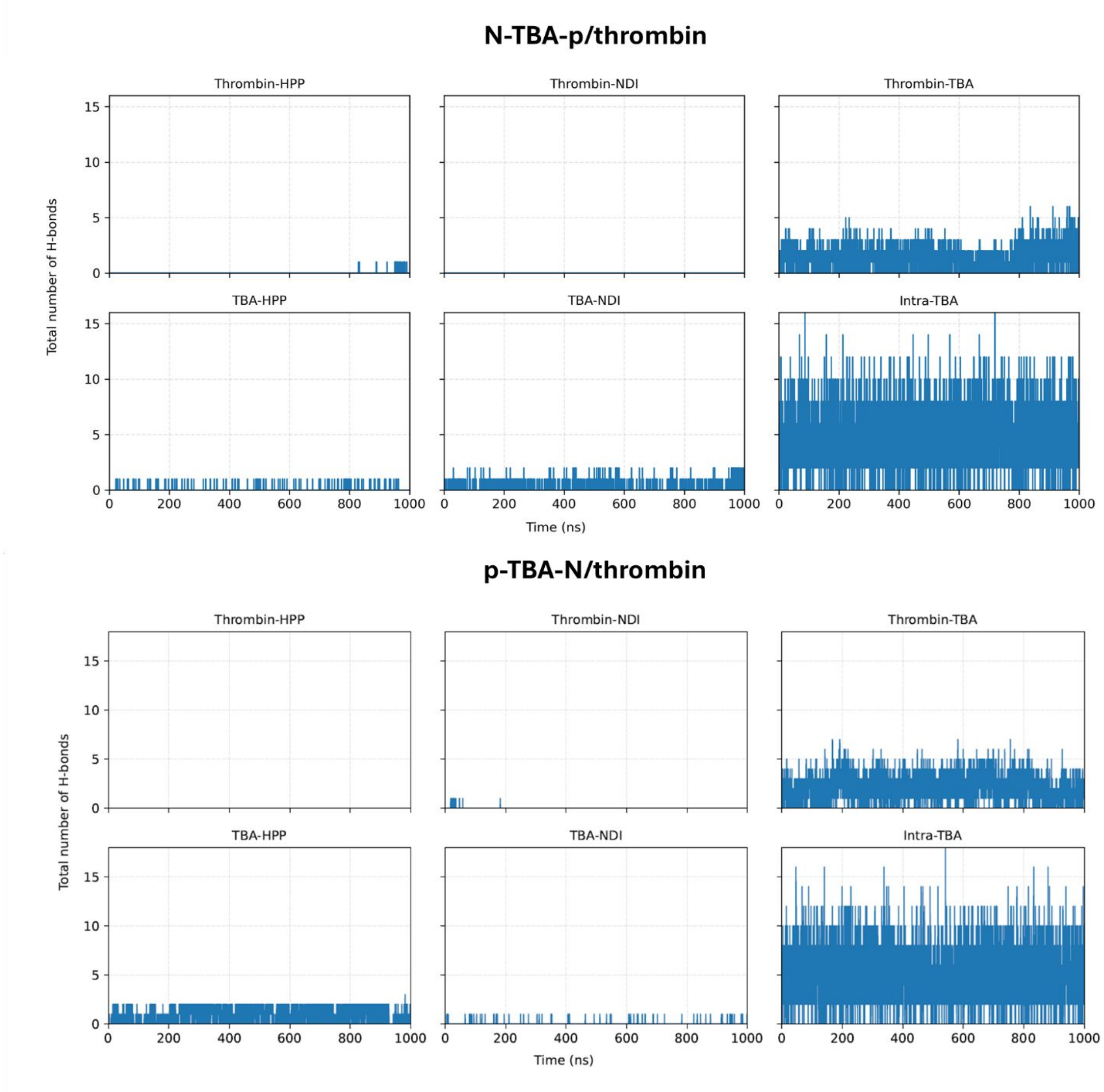

Figure S7. Total number of hydrogen bonds calculated on the 1 μs MD simulations performed for **N-TBA-p**/thrombin and **p-TBA-N**/thrombin (replica 1 of each system). NDI = naphthalene diimide; HPP = 3-hydroxypropylphosphate.
